# Taiwan ended third COVID-19 community outbreak as forecasted

**DOI:** 10.1038/s41598-024-56692-0

**Published:** 2024-03-19

**Authors:** Yu-Heng Wu, Torbjörn E. M. Nordling

**Affiliations:** Department of Mechanical Engineering, National Cheng Kung University, Tainan, 701 Taiwan

**Keywords:** Computational models, Infectious diseases, Computational models, Infectious diseases

## Abstract

Accurate forecasting of community outbreaks is crucial for governments to allocate healthcare resources correctly and implement suitable non-pharmaceutical interventions. Additionally, companies must address critical questions about stock and staff management. Society’s key concern is when businesses and organizations can resume normal operations. Between December 31st 2019 and 2021, Taiwan experienced three separate COVID-19 community outbreaks with significant time intervals in between, suggesting that each outbreak eventually came to an end. We identified the ratio of the 7-day average of local & unknown confirmed to suspected cases as the key control variable and forecasted the end of the third outbreak by the exponential model. We forecasted the end of the third outbreak on Aug. 16th with threshold ratios of $1.2\cdot 10^{-4}$. The real observations crossed the threshold on Aug. 27th, eleven days later than forecasted, with the last case of the third outbreak confirmed and quarantined on Sept. 20th. This demonstrated the accuracy of the proposed forecasting method in predicting the end of a local outbreak. Furthermore, we highlight that the ratio reflects the effectiveness of contact tracing. Effective contact tracing together with testing and isolation of infected individuals is crucial for ending community outbreaks.

## Introduction

**Figure 1:**
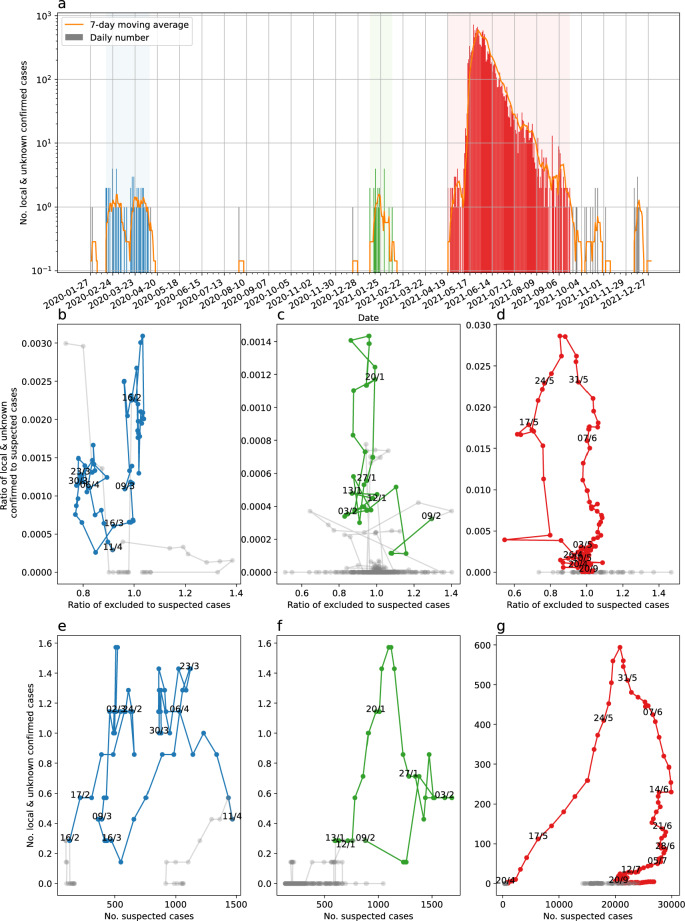
Reported number of local and unknown infected and 7-day moving average in Taiwan from the first local case 2020-01-28 until 2021-12-31 (**a**). The three community outbreaks are marked in blue (1st), green (2nd), and red (3rd). The cases between are not connected. The ratio of local and unknown confirmed to suspected cases versus ratio of excluded to suspected cases reported for the three outbreaks (**b**–**d**). Number of local and unknown confirmed cases versus number of suspected cases reported for the three outbreaks (**e**–**g**). The 7-day moving average of each quantity is used in (**b**–**g**).

In Taiwan, the first outbreak of COVID-19 started before February 16th and ended on April 11th, 2020 (54 days), see Fig. 1a. The second outbreak started before January 12th and ended on February 9th, 2021 (27 days). The third outbreak started before April 20th and ended on September 20th, 2021 (154 days). The cases reported in December were infected by new cases from abroad having no relation to the fourth outbreak in 2022 and were quickly controlled by Taiwan Center for Disease Control (CDC)^1^. Between the first and second outbreaks, Taiwan saw a period of 273 days without any reported local infection, excluding an isolated case of unknown source reported on August 2nd and a local case reported on December 22nd. Between the second and third outbreaks, Taiwan had 69 days without any reported local infected cases. Between the third and fourth outbreaks, Taiwan had 104 days without any reported local infected cases, with the exception of 22 old cases reported in September and October. The cases reported in December were infected by newly abroad cases having no relation to the fourth outbreak and were quickly controlled by Taiwan CDC.

The third outbreak had a significant impact on the retail catering market in Taiwan. According to the Department of Statistics, the revenue growth rate of the retail market dropped to -13.3$\%$ in June 2021 and remained negative until September^2^. For the catering market, the revenue growth rate was negative since May and dropped to -39.9$\%$ in June and remained negative until October. The unemployment rate also increased from around 3.7$\%$ in April to 4.8$\%$ in June, before decreasing back to 3.66$\%$ in November. The number of people suffering unpaid leave also increased significantly, from 4125 in May to 13,626 in June, and continued to rise to 58,731 in August, before decreasing to 18,253 in December.

Several researchers attempted to forecast the COVID-19 end date using various models, including the Weibull distribution model, ARIMA model, SIR model, and stochastic model^3–6^. Chaturvedi et al. implemented the SIR model and estimated the end of COVID-19 under different vaccination conditions^5^. Such a comparison helps the government allocate resources to reduce economic costs and expedite the COVID-19 pandemic end date. However, all of these forecasts were solely based on the number of confirmed cases or deaths. A comparison between the forecasting results and the actual data was not made. In this paper, we focus on the ratio of the 7-day average of local & unknown confirmed to suspected cases as the key control variable and forecast the end of the third outbreak using the exponential model. We also compare the forecasted end date with the actual data.

## Results

On June 27th 2021, we published a preprint paper forecasting the end of the outbreak^7^. In short, the ratio of the 7-day moving average of confirmed infected to suspected has already fallen to less than one-third of the peak value of 0.029 on May 28th, while the number of daily suspected has increased to above 25 000, and the ratio of the 7-day moving average of excluded to suspected was slightly above one, which is needed to resolve the backlog from when it was down at 0.37. In other words, the contact tracing, testing, and isolation of infected had, after initial failure and challenges, successfully been scaled up to the required volume of around 25 000 suspected per day and the number of confirmed infected was decreasing. The same pattern was repeated during all three outbreaks: first, the number of confirmed infected increase rapidly and the contact tracing, testing, and exclusion of cases lagged, then focus on contact tracing brought up the number of suspected and investment into expanding testing cleared the backlog, see Fig. 1b–g.

**Figure 2:**
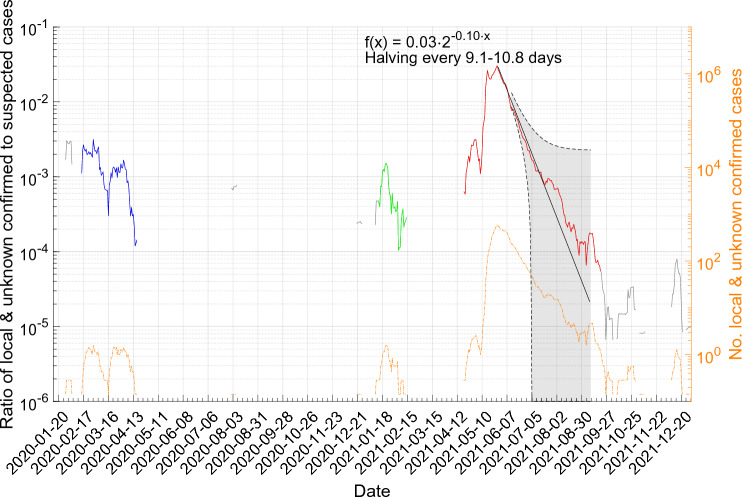
Evolution of the ratio of the 7-day moving mean of local & unknown confirmed to the 7-day moving mean of suspected cases and our forecast (black solid line) of the end of the third outbreak with the 95% confidence bounds (grey shaded area). The actual data of the three community outbreaks are marked in blue (1st), green (2nd), and red (3rd). The cases between in grey are not connected. The orange dash-dotted line is the 7-day moving average number of local and unknown confirmed cases, identical to the orange line in Fig. 1a.

We used an exponential model to fit the daily ratios of the 7-day moving average of confirmed infected to suspected cases, which decreased from a peak of 0.029 on May 28th to 0.0077 on June 16th. The calculated non-simultaneous prediction bounds on a new observation with 95% confidence, drawn as black dashed lines in Fig. 2, puts the earliest end date on July 2nd and the latest indefinitely into the future. The latter is in agreement with a failure to execute the contact tracing, testing, and isolation of new local clusters emerging from people infected abroad.

The pandemic was in a stage with halving of the ratio every 9.9 days (95% confidence bounds 9.1 - 10.8 days). The previous two outbreaks ended after the ratio was brought down to $1.2\cdot 10^{-4}$, see Fig. 2, so we assumed that the same was required to end this outbreak. Note that the length of the window of our moving average and the baseline number of suspected affect this ratio. When only one confirmed infected remains, the nominator will be 0.14, i.e. 1/7, and the denominator then needs to be around 1200, i.e. on average 1200 suspected each day for the last confirmed infected. The mean 7-day moving average number of suspected between the 1st and 2nd outbreak was 324 and between the 2nd and 3rd outbreak it was 684.

By extrapolation, we forecasted that this ratio would be reached on 2021-08-16 after the required six halvings. While the real observation exceeded the threshold on 2021-08-27 and decreased until 2021-09-26. In addition, Taiwan CDC suspected that cases after 2021-09-20 were old cases. This forecast depends on contact tracing, testing, and isolation being equally effective as during the past three weeks and no new local cluster emerging from people infected abroad.

Both to evaluate and illustrate the behaviour of our exponential model applied to the ratio of local and unknown confirmed to suspected cases, we in the supplementary, for all three outbreaks, report on the impact of changing the end date of the period used to fit the model, as well as a fixed 14-day fitting period with varying starting dates (supplementary Fig. 4). Moreover, to check the impact of corrections to the data published by Taiwan CDC after our original forecast, we refitted the ratio of local and unknown confirmed to suspected cases of the updated data to forecast the end of the third COVID-19 outbreak. This updated forecast differs by a mere three days from the original one (supplementary Fig. 3). For comparison, we also fitted the classic Richards model^8^ to the cumulative number of local and unknown confirmed cases. It yielded an overly optimistic forecast of the end of the pandemic already in mid-July (supplementary Fig. 5).

## Discussion

From June 2020 until the third outbreak, life in Taiwan was pre-pandemic, except for the mandatory mask and temperature checks in the public transportation introduced on March 31st, 2020 and at large events^9^. Large events with tens of thousands of visitors were held, such as the Mayday New Year’s Eve pop concert at Taoyuan International Baseball Stadium on December 31st, 2020^10^. Both the 2nd and 3rd outbreaks involved the Alpha variant (B.1.1.7), which Hsu et al.^11^ estimated to have a 1.44-fold higher infection probability and 57% higher basic reproduction number based on household transmissions during the 1st and 2nd outbreak, in agreement with previous estimates of 43–90%^12^. Evidently, the measures in place to reduce transmissibility and prevent a large-scale outbreak were not sufficient, so Taiwan should have had a surge in cases of community transmission existed during the two periods without local infections. In addition, the National Health Insurance Administration (NHIA) was proactively seeking out patients with severe respiratory symptoms in its database and allowing all hospitals, clinics, and pharmacies to see the patients’ travel history obtained from the National Immigration Agency from February 18th, 2020 onwards^13^. The third outbreak has been traced to a cluster of infected China Airline pilots and the Novotel at Taoyuan International Airport violating the quarantine rules by housing quarantined flight crews and local guests in the same building^14,15^. This initial failure of the mandatory quarantine at the border and contact tracing during the third outbreak combined with the success of contact tracing during the 2nd and 3rd outbreaks and the transmissibility reduction measures remaining the same, implies that Taiwan’s success was due to border control and tracing of contacts upon suspicion. Thus we can with confidence say that Taiwan ended the three community outbreaks thanks to contact tracing, testing, and isolation. This leads us to the conclusion that Taiwan serves as an exemplary case, implementing a near ideal non-pharmaceutical intervention (NPI).

The Taiwanese COVID-19 control strategy implemented by the Central Epidemic Command Center (CECC), activated on January 20th, 2020^13^, is based on six main pillars: (a) border control with quarantine upon arrival, (b) self-health monitoring when having visited a place with known cases, (c) testing when showing symptoms and seeking medical care, (d) mandatory supervised quarantine of confirmed infected and individuals at high risk of having been infected, (e) contact tracing, and (f) a four-level system of measures to suppress community spreading. These are devised to control the effective reproduction number, i.e. the expected number of people an infected individual will transmit the disease to while infectious. The reproduction number can be seen as the transmission risk per contact (transmissibility), times the number of contacts per day, and times the number of days the person is infectious. From June 7th until May 11th, 2020, no restrictions on the size of gatherings, i.e. curbing of the number of contacts, existed^15,16^. Taiwan had a system with four levels of non-pharmacological interventions (NPIs). The level 3 measures in place from May 19th, 2021 until July 27th, 2021 consist of mandatory wearing of masks at all times outside private spaces and social distancing, which reduce the transmissibility; indoor gatherings limited to five people and closure of certain businesses, partly including schools and preschools, which reduce the number of contacts; and mandatory COVID-19 testing in areas where community transmission has taken place, which reduces the number of days an infected person can transmit the disease before being quarantined^17–19^. As demonstrated by numerous countries, such as the United Kingdom and United States, community suppression measures, such as lockdowns, alone do not end a community outbreak.

The key to ending an outbreak is to focus on contact tracing to bring up the number of suspected and increase testing and isolation/care capacity. The focus on contact tracing and bringing up the number of suspected is key because one needs to prevent every infected person from infecting a new person to end the outbreak. It makes the ratio of confirmed infected to suspected the essential metric to follow. The Lancet Commission Task Force on public health measures to suppress the pandemic also report on the effectiveness of contact tracing and quarantine measures, albeit not on its use to end outbreaks^20^. Since May 19th, 2021, Taiwan has implemented an SMS text message contact tracing system based on each person upon entry in each business scanning a place specific QR code that generates a unique SMS sent to 1922–the Taiwan Center for Disease Control hotline, which helps scale the contact tracing^21^.

Using the exponential model to fit the 7-day average of local & unknown confirmed to suspected cases, we obtained a forecast that differed by only 11 days from the real observation crossing the threshold, showing that our method can predict the end date of a community outbreak when the policy remains unchanged. Taiwan ended the three community outbreaks thanks to contact tracing, testing, and isolation. In addition to forecasting outbreak endpoints, we posit that our model can serve as a metric for assessing the alignment of an outbreak with the projected trend, indicative of effective adherence to an ideal Non-Pharmaceutical Intervention (NPI). We specifically emphasize its utility as a metric for gauging the effectiveness of contact tracing-observing that an increase in the number of suspected cases relative to confirmed cases is crucial for ending an outbreak. As illustrated in supplementary Fig. 4, our model forecasts that the outbreak will continue indefinitely until the contact tracing has been scaled up so the number of suspected cases enables capture of infected cases. The dual functionality of our model positions it as a valuable tool for governments, also aiding in the discernment of the optimal timing for easing stringent NPI measures, such as lockdowns.

After this report on how Taiwan ended three outbreaks, we hope the world would take notice and learn from the Taiwanese strategy, so unnecessary suffering and deaths can be avoided in future pandemics. Like Wang et al.^13^, we think “Taiwan is an example of how a society can respond quickly to a crisis and protect the interests of its citizens.”

## Methods

To limit the influence of reporting differences between the weekdays and fluctuations in when cases are reported to the Taiwan CDC, we use the 7-day moving average calculated over the past seven days. It is worth noting that our moving average covers the mean serial interval, which was estimated to be 6.2 days among the 42 infector-infectee pairs during the 1st outbreak^22^.

To forecast when the third community outbreak will end, we assumed that the decrease in the ratio of local & unknown confirmed to suspected cases can be described as an exponential growth process. More precisely, we assumed the model

1 $$\begin{aligned} f(x) = a\cdot 2^{-b\cdot x}, \end{aligned}$$

where *a* is the initial value and the reciprocal of *b* is the half-time. We fitted this model to the ratio of the 7-day moving average of local & unknown confirmed to the 7-day moving average of suspected cases from the peak on 2021-05-28 to 2021-06-16, i.e. 19 time points, using the Curve Fitting Toolbox in Matlab R2022b by MathWorks using the “NonlinearLeastSquares” estimator. The root mean squared error of the model fitted to the data points was 0.0011 and adjusted R-squared 0.97, indicating a good fit that explains the data well. The estimated parameters are *a* 0.029 (95% confidence bounds 0.028–0.030) and *b* 0.10 (95% confidence bounds 0.09–0.11).

All data used is for the readers convenience also included in the supplementary Table 4, 5, 6 and 7. The data preprocessing is illustrated in supplementary Figs. 1 and 2. For comparison we also fitted the classic Richards model^8^ to the cumulative number of local and unknown confirmed cases, see supplementary.

### Supplementary Information

Supplementary Information.

## Data Availability

All data is publicly available and was collected from the Taiwan Centers for Disease Control (Taiwan CDC). We collected the cumulative number of confirmed infected with SARS-CoV-2 with a known local source and unknown source, as well as the cumulative reported suspected and excluded cases, from the Taiwan CDC News Bulletins, available in Chinese at https://www.cdc.gov.tw/Bulletin/List/MmgtpeidAR5Ooai4-fgHzQ (last visited 2022-05-23).
