# Supplementary material for: Taiwan ended third COVID-19 community outbreak as forecasted
Source: Sci Rep. 2024 Mar 19;14:6596. doi: 10.1038/s41598-024-56692-0 (PMC10951404; doi:10.1038/s41598-024-56692-0)
Supplement: Supplementary file 1 — Supplementary Information. [file 41598_2024_56692_MOESM1_ESM.pdf]

Contents

1 Data collection 1

2 Data cleaning and preprocessing 1

3 Refitting to the updated data, 2023-03-08 2

4 Multiple prediction cycles 3

5 Comparison with other models 4

6 Data used 4

Supplementary information

Here we provide details on the data collection, cleaning, and analysis to enable verification and reproduction of the results.

1 Data collection

All data is publicly available and was collected from the Taiwan Centers for Disease Control (Taiwan CDC). We collected the cumulative number of confirmed infected with SARS-CoV-2 with a known local source and unknown source, as well as the cumulative reported suspected and excluded cases, from the Taiwan CDC News Bulletins, available in Chinese at `https://www.cdc.gov.tw/Bulletin/List/MmgtpeidAR5Ooai4-fgHzQ` (last visited 2022-05-23). Unfortunately, these news bulletins have not been issued every day, nor at the same time of the day throughout the pandemic, which means that the daily increase derived by subtracting the value in the previous report is influenced by the varying time between these reports. The way of counting the number of suspected and excluded was changed on 2020-03-06, which contains a large correction to these cumulative numbers. We therefore also downloaded the "Daily Number of Cases Suspected SARS - CoV-2 Infection Tested" dataset from the Taiwan CDC Open Data portal `https://data.cdc.gov.tw/en/dataset/daily-cases-suspected-sars-cov-2-infection_tested` (last visited 2021-06-17). This dataset contains the daily number of cases suspected to have SARS-CoV-2 from three sources: (i) notifications of infectious diseases, (ii) home quarantine and inspection, and (iii) expanded monitoring, as well as the total daily number.

2 Data cleaning and preprocessing

According to Taiwan CDC News Bulletins, cases 16821, 16841, 16851, 16856, 16862, and 16865 were wrongly reported as abroad positive cases and CDC updated their local positive confirmed date on 2021-12-17. However, 2021-12-17 is not their real positive test date. Therefore, we have corrected the abroad positive dates as the local positive dates and removed the 7 confirmed cases on 2021-12-17.

We removed the first CDC News Bulletin for the days when more than one was issued, thus keeping the later report with the larger cumulative numbers. Then we corrected the number of days in between the report for all cases with more than one day in between by evenly distributing the change in cumulative number over the days in between and ensuring that the cumulative sum equals the reported one. Due to the change in the way of counting the number of suspected and excluded on 2020-03-06, the number of suspected cases is unreasonably low before it and a jump correcting for the change occur on that day. These artefacts are clearly seen in Figure 1 as a dip in the number of suspected before and a value slightly above 10 000 on this day. These artefacts clearly influence the 7-day moving average of the number of suspected that we need for getting a stable estimate of the ratio of confirmed infected to suspected cases. The total daily number of suspected cases in the CDC Open Data Portals dataset does not contain this error, so we decided to use it instead.

Since the number of excluded cases only was available in the CDC News Bulletins and contain a similar change in the way of counting on 2020-03-06, we assumed that the ratio of excluded to suspected was correct and used the ratio to estimate the number of daily excluded based on the total number of daily suspected from the CDC Open Data dataset. To ensure that the cumulative sum of daily excluded matched the reported values in the News Bulletins after March 6th, we added or removed excluded cases proportionally to the daily suspected from the days between two reports iteratively from the later report day to the former to avoid errors from rounding to whole integers later. This resulted in an estimate of the daily total number of excluded cases that on each day with a CDC News Bulletin report match the reported number exactly. The result of our preprocessing is visualised in Figure 1, where the number of suspected cases from the corrected News Bulletin and Open Data closely match after 2020-03-06. Note that a red circle without a dot in it indicate that more than one day had passed between the consecutive News Bulletins and the change is therefore larger than expected from one day to the next. We have verified that the cumulative number of excluded always remains smaller than the cumulative number of suspected.

Since our analysis is dependent on the 7-day moving average of suspected and excluded we also evaluated the relative difference in the 7-day moving average daily values obtained from the CDC News Bulletin and Open Data, relative to the

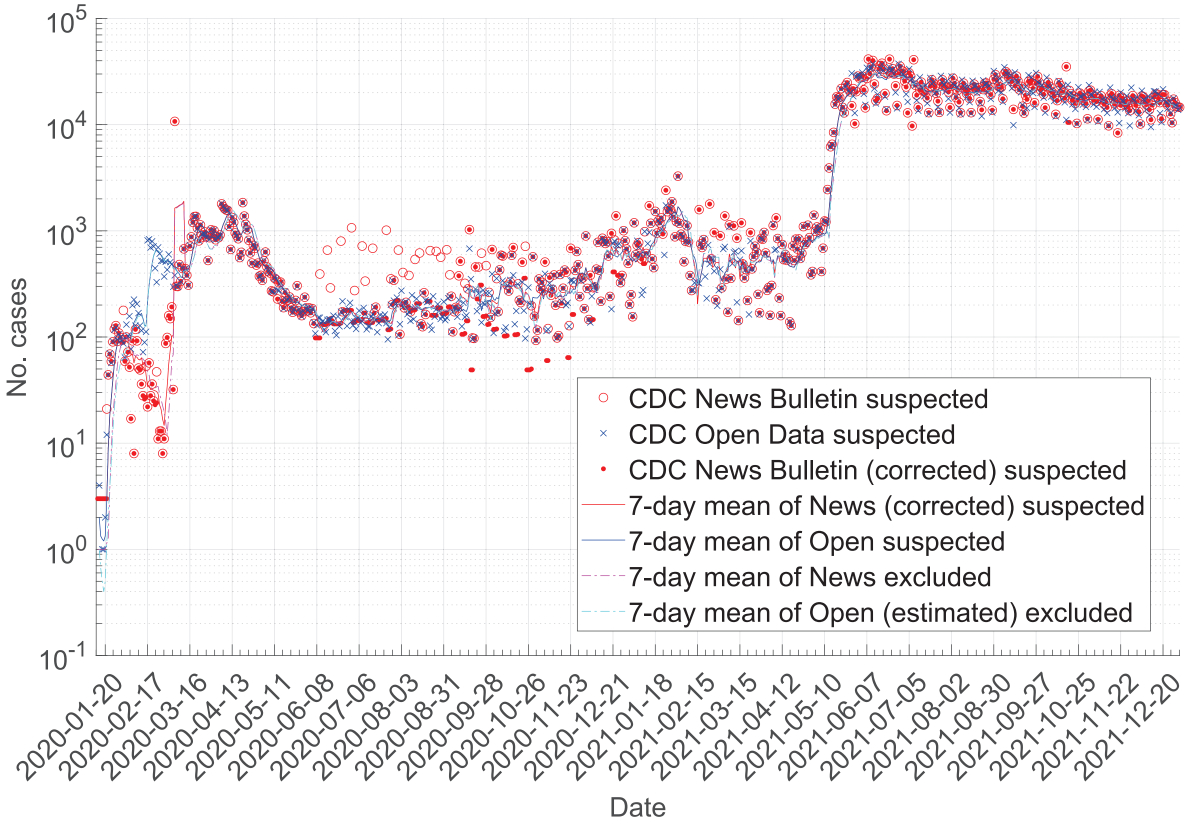

Figure 1. Comparison of the change in number of suspected between CDC News Bulletins (red circles), the total daily number of suspected in the CDC Open Data (blue x), and the change in number of suspected between CDC News Bulletins corrected for the number of days in between (red dot), and the 7-day moving average of the later two (blue and red lines), and the 7-day moving average of the change in number of excluded between CDC News Bulletins corrected for the number of days in between (magenta line), and the 7-day moving average of our estimate of the daily number of excluded based on the total daily number of suspected in the CDC Open Data (cyan line)

latter. The relative difference in number of suspected remains below 41% every day after 2020-03-06, below 10% 81% of the days, and below 1% 33% of the days. The large relative differences occur on separated days with small relative differences in between and are caused by differences in on which day extreme numbers of suspected are reported, see Figure 2. This is to be expected, since the Open Data most probably contains all suspected cases on each day, while the News Bulletins contain the cases added since the last bulletin. Thus these differences may shift values by one day, but no more. We cannot tell which of these two data sources is more reliable, but considering that the Open Data at least is corrected for the change in the way of calculating the number of suspected we decided to use it and our estimate of the total daily number of excluded based on it. This does not affect the ratio of excluded suspected cases, but it affects the ratio of local & unknown confirmed to suspected cases by up to 41% on individual days, which in practice means that specific values may be shifted in time by one day. Since our forecast of when the third outbreak will end is based on 19 time points any effects of this difference are well within the estimated confidence intervals.

3 Refitting to the updated data, 2023-03-08

The Taiwan CDC has made minor changes to the data, so we repeated the data cleaning and refit the exponential model to the cleaned data. All results are similar to our previous prediction and the forecasting result is shown in Figure 3. The forecasted end date of the third outbreak is on Aug. 13th with threshold ratio of $1.2 * 10^{-4}$ The predicted half-time is 10 days (95% confidence interval: 8.9-10.4). We fitted this model to the ratio of the 7-day moving average of local & unknown confirmed to the 7-day moving average of suspected cases from the peak on 2021-05-27 to 2021-06-16, i.e. 21 time points. The estimated parameters are a 0.030 (95% confidence bounds 0.029 - 0.031) and b 0.10 (95% confidence bounds 0.096 - 0.11).

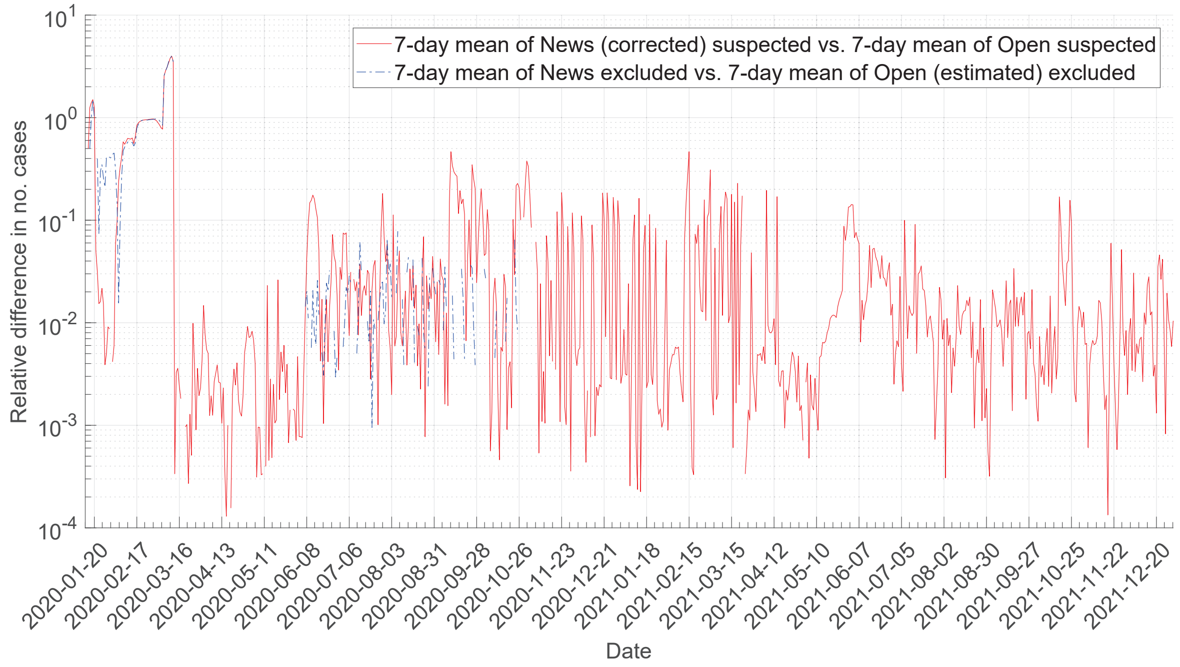

Figure 2. Relative difference in the 7-day moving average of the change in number of suspected between CDC News Bulletins corrected for the number of days in between and the 7-day moving average of the total daily number of suspected in the CDC Open Data (red line), and the 7-day moving average of the change in number of excluded between CDC News Bulletins corrected for the number of days in between and the 7-day moving average of our estimate of the daily number of excluded based on the total daily number of suspected in the CDC Open Data (magenta line). No line indicates that the difference is zero

4 Multiple prediction cycles

Since we operate on the ratio of local confirmed to suspected cases and we assume the policy, i.e. NPI, to remain the same, our forecasts are only valid as long as this assumption holds. During each outbreak in Taiwan, the contact tracing and testing effort was ramped-up, i.e. a change in policy happened during each outbreak. Since we did not know at what pace this ramp-up was going to occur, we considered it best to do each forecast under the assumption of the policy being unchanged. This is so that our forecasts show a continued outbreak until the moment when the ramp-up is sufficient to end the outbreak. We consider it important to maintain a sense of urgency so there is motivation to change the policy until the policy is sufficient to end the outbreak so that the forecasts contribute to the right actions being taken. This implies that the forecasting needs to be updated every few days and the latest forecast is always the most accurate. Using a shorter interval for the training means that the model quicker picks up on changes in policy but also becomes more sensitive to noise in the data and random events.

We fit the exponential model with a fixed 14-day moving interval with a stride of 3 days and forecast the ratio of the future 3 weeks. The results are shown in Figure 4. The fitting result of the third outbreak is starting upward since it is in the increasing trend, so the forecast goes up. It is until the peak time, May 27th, 2021, the forecasting result starts giving a negative slope and shows a strong indication of a decreasing trend.

To determine the minimum fitting period, we consider the initiation of our fitting process when the ratio begins to decline. Effectively mitigating the outbreak requires an increase in contact tracing capacity. This capacity could be assessed by examining the ratio of excluded to suspected cases, as depicted in Figure 1d. The ratio initially dips below 1 at the onset of the outbreak and rises above 1 again on June 6, 2021. As this ratio gets close to 1, it means we're nearing a normal level of contact tracing capacity. We investigated the influence of adjusting the data fitting period, forecasting the ratio and determining the date on which the fitted line crosses the threshold. The comparison between the forecasted end date for the third outbreak and the actual threshold passing date of August 27th, 2021 is presented in the accompanying Table. 1. The R^2 consistently remains above 0.95. The absolute difference in days between the true end date and our forecasted end date remains below 14 days when considering the fitting period from May 27th to June 4th or beyond. The ratio of excluded to suspected cases remains close to 1 indicating a normal state of contact tracing capacity. Furthermore, we also forecasted the end of the first and second outbreaks by changing the fitting period. The results are presented in Table 2 and 3.

Table 1. Forecasting the end of the third outbreak keeping the start date fixed on May 27th and varying the end date of the fitting interval from May 30, 2021, to June 15, 2021. The actual data crossed the threshold we used to define the outbreak end on August 27th, 2021.

Fitting period	Forecasted outbreak end date	Days from true end date	Ratio of Excluded to Suspected Cases on the End Date of Fitting	R^2
5/27 to 5/30	8/6	-21	0.94	0.95
5/27 to 5/31	8/5	-22	0.96	0.97
5/27 to 6/1	8/7	-20	1.03	0.98
5/27 to 6/2	8/9	-18	1.04	0.99
5/27 to 6/3	8/9	-18	1.06	0.99
5/27 to 6/4	8/13	-14	1.06	0.98
5/27 to 6/5	8/19	-8	1.01	0.96
5/27 to 6/6	8/22	-5	1.01	0.96
5/27 to 6/7	8/24	-3	1.00	0.96
5/27 to 6/8	8/24	-3	1.02	0.97
5/27 to 6/9	8/22	-5	0.98	0.97
5/27 to 6/10	8/20	-7	0.98	0.97
5/27 to 6/11	8/17	-10	1.00	0.97
5/27 to 6/12	8/15	-12	1.01	0.97
5/27 to 6/13	8/13	-14	1.03	0.97
5/27 to 6/14	8/13	-14	1.06	0.97
5/27 to 6/15	8/13	-14	1.02	0.98
**5/27 to 6/16**	**8/13**	**-14**	**1.06**	**0.98**

5 Comparison with other models

During the escalation of the third outbreak, Taiwan's CDC ceased the daily reporting of recovery numbers. Specifically, there is a gap in recovery data from May 20th, 2021, to June 13th, 2021. Unfortunately, the 7-day moving average time data utilized for fitting our exponential model spans from May 27th, 2021, to June 16th, 2021, which largely overlapes the gap in the recovery data, creating challenges in comparing the SIR type model with our exponential model.

We thus implement the classic Richards model.The Richards model is described by the ordinary differential equation (ODE):

$$\frac{dC(t)}{dt} = r \cdot C(t) \cdot \left(1 - \left(\frac{C(t)}{k}\right)^{\alpha}\right), \tag{1}$$

where $C(t)$ is the daily cumulative number of confirmed cases, r is the growth rate, k is the carrying capacity, and α governs the shape of the growth curve. The parameter values obtained from the fitted result are $r = 2.9 \times 10^{-5}$, $k = 1.24 \times 10^4$, and $\alpha = 5.3 \times 10^{-5}$.

To facilitate a comparison with our exponential model, we transformed our forecasting of the ratio of local confirmed cases to suspected to daily confirmed cases. This transformation involved multiplying the ratio by the average value of the suspected cases in our fitting period (May 27 to June 16). The comparison result is shown in Figure 5. For the Richards model, we fitted the data from April 20 to June 16, marked as the magenta-shaded area. Our results better align with the actual data (red line) compared to the Richards model.

6 Data used

The data we used for forecasting is included in Table 4-7. The updated data we used for refitting is shown in 8-12.

Table 2. Forecasting the end of the first outbreak keeping the starting date fixed on February 28th and varying the end date of the fitting interval from March 3rd to April 13th, 2020. The actual data crossed the threshold ratio on April 15th, 2020.

Fitting period	Forecasted outbreak end date	Days from true end date	Ratio of Excluded to Suspected Cases	R^2
2/28 to 3/2	3/28	-18	1.01	0.75
2/28 to 3/3	4/1	-14	0.99	0.80
2/28 to 3/4	4/14	-1	0.97	0.50
2/28 to 3/5	5/9	24	0.96	0.16
2/28 to 3/6	4/15	0	0.96	0.44
2/28 to 3/7	4/10	-5	0.98	0.58
2/28 to 3/8	4/7	-8	0.99	0.67
2/28 to 3/9	4/5	-10	0.96	0.73
2/28 to 3/10	4/5	-10	0.99	0.76
2/28 to 3/11	4/4	-11	0.99	0.80
2/28 to 3/12	4/3	-12	1.00	0.83
2/28 to 3/13	4/3	-12	1.00	0.85
2/28 to 3/14	4/2	-13	0.98	0.86
2/28 to 3/15	4/2	-13	0.99	0.87
2/28 to 3/16	4/2	-13	0.92	0.88
2/28 to 3/17	4/2	-13	0.85	0.88
2/28 to 3/18	4/3	-12	0.80	0.87
2/28 to 3/19	4/5	-10	0.77	0.84
2/28 to 3/20	4/7	-8	0.78	0.82
2/28 to 3/21	4/9	-6	0.77	0.76
2/28 to 3/22	4/12	-3	0.79	0.70
2/28 to 3/23	4/15	0	0.79	0.65
2/28 to 3/24	4/17	2	0.80	0.62
2/28 to 3/25	4/20	5	0.80	0.57
2/28 to 3/26	4/22	7	0.81	0.56
2/28 to 3/27	4/26	11	0.81	0.51
2/28 to 3/28	4/29	14	0.84	0.49
2/28 to 3/29	5/2	17	0.77	0.47
2/28 to 3/30	5/7	22	0.78	0.41
2/28 to 3/31	5/12	27	0.78	0.37
2/28 to 4/1	5/17	32	0.84	0.34
2/28 to 4/2	5/25	40	0.85	0.28
2/28 to 4/3	6/2	48	0.84	0.24
2/28 to 4/4	6/8	54	0.84	0.22
2/28 to 4/5	6/14	60	0.89	0.21
2/28 to 4/6	6/15	61	0.83	0.22
2/28 to 4/7	6/17	63	0.85	0.24
2/28 to 4/8	6/18	64	0.87	0.26
2/28 to 4/9	6/16	62	0.88	0.29
2/28 to 4/10	6/13	59	0.90	0.32
2/28 to 4/11	6/11	57	0.92	0.35
2/28 to 4/12	6/9	55	0.96	0.37
2/28 to 4/13	6/7	53	1.14	0.40

Table 3. Forecasting the end of the second outbreak keeping the starting date fixed on January 22nd and varying the end date of the fitting interval from January 25th to February 5th, 2021. The actual data crossed the threshold ratio on February 5th, 2021.

Fitting period	Forecasted outbreak end date	Days from true end date	Ratio of Excluded to Suspected Cases	R^2
1/22 to 1/25	2/7	2	0.99	0.69
1/22 to 1/26	2/5	0	0.98	0.81
1/22 to 1/27	2/6	1	0.95	0.85
1/22 to 1/28	2/4	-1	0.93	0.91
1/22 to 1/29	2/5	0	0.91	0.87
1/22 to 1/30	2/6	1	0.88	0.88
1/22 to 1/31	2/6	1	0.97	0.89
1/22 to 2/1	2/7	2	0.93	0.88
1/22 to 2/2	2/7	2	0.83	0.88
1/22 to 2/3	2/8	3	0.85	0.88
1/22 to 2/4	2/9	4	0.95	0.84
1/22 to 2/5	2/9	4	1.10	0.86

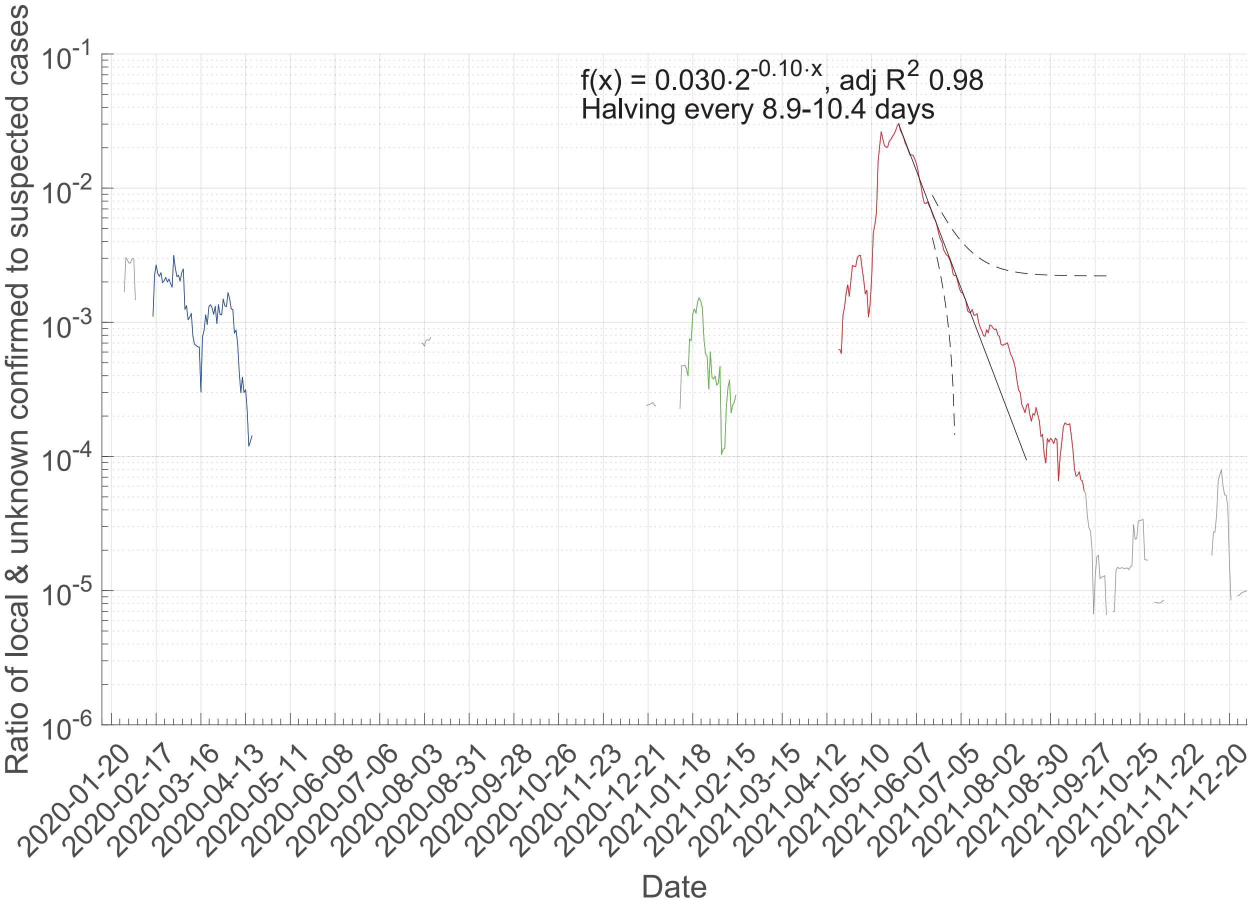

Figure 3. Refit of the forecast of the ratio of the 7-day moving average of local & unknown confirmed to the 7-day moving average of suspected cases and our forecast of the end of the third outbreak. The three community outbreaks are marked in blue (1st), green (2nd), and red (3rd). The cases between in grey are not connected

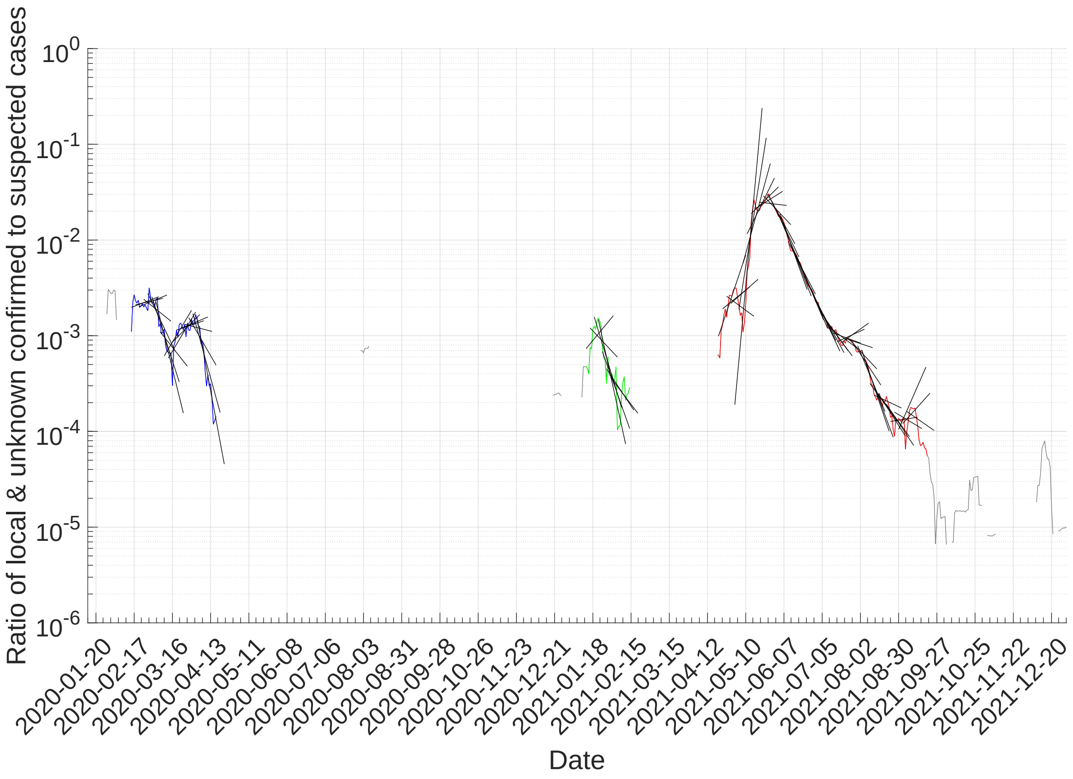

Figure 4. Forecasts of the exponential model (black solid lines) with 14-day moving window with a stride of 3 days. The three community outbreaks are marked in blue (1st), green (2nd), and red (3rd). The cases between in grey are not connected.

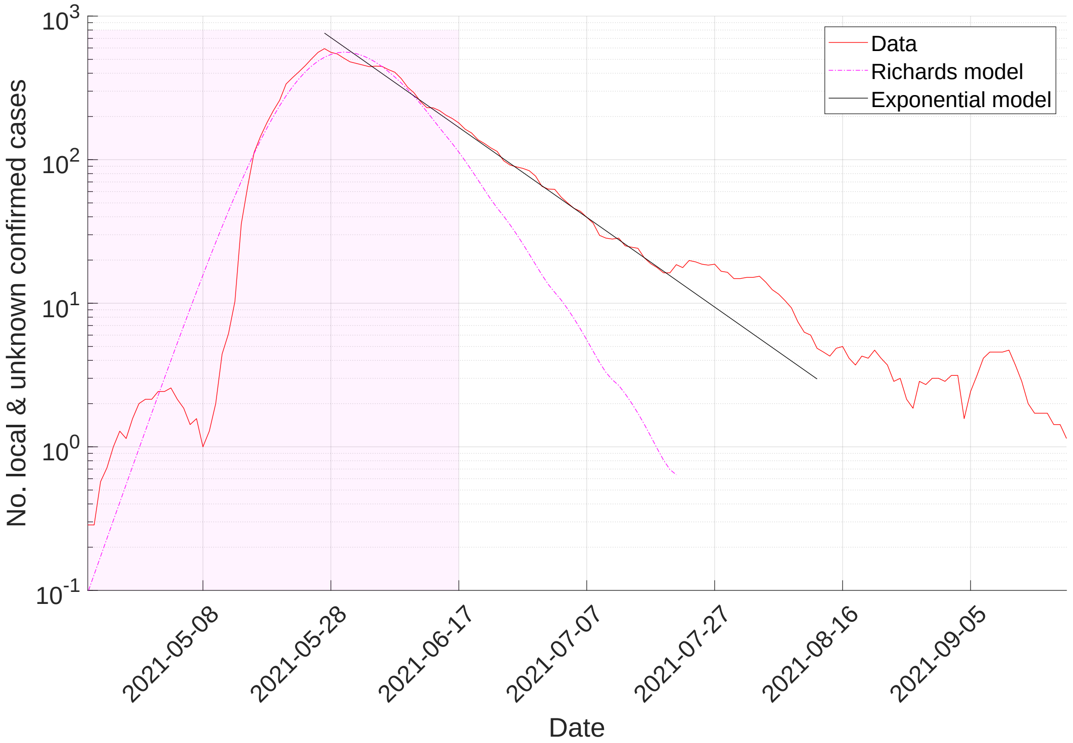

Figure 5. Comparison of the forecast results of the exponential model (black solid line) and the Richards model (magenta dash-dotted line). The three community outbreaks are marked in blue (1st), green (2nd), and red (3rd). The cases in grey are not connected. The magenta-shaded area is the time period for fitting the Richards model.

Table 4. The data used for forecasting

Date	Susp.	Excl.	Conf.	Date	Susp.	Excl.	Conf	Date	Susp.	Excl.	Conf
2020-01-15	0	0	0	2020-03-03	506	466	0	2020-04-20	1835	931	0
2020-01-16	4	2	0	2020-03-04	485	454	1	2020-04-21	1371	1526	0
2020-01-17	0	0	0	2020-03-05	297	283	1	2020-04-22	1154	1469	0
2020-01-18	1	0	0	2020-03-06	381	393	0	2020-04-23	1014	1163	0
2020-01-19	1	0	0	2020-03-07	307	382	0	2020-04-24	827	1132	0
2020-01-20	2	1	0	2020-03-08	298	313	0	2020-04-25	619	1091	0
2020-01-21	12	5	0	2020-03-09	476	359	1	2020-04-26	495	738	0
2020-01-22	44	7	0	2020-03-10	332	376	0	2020-04-27	740	588	0
2020-01-23	70	14	0	2020-03-11	445	395	0	2020-04-28	694	792	0
2020-01-24	57	20	0	2020-03-12	677	685	1	2020-04-29	484	705	0
2020-01-25	91	42	0	2020-03-13	439	454	0	2020-04-30	495	513	0
2020-01-26	117	58	0	2020-03-14	379	405	0	2020-05-01	374	477	0
2020-01-27	123	64	0	2020-03-15	306	341	0	2020-05-02	380	436	0
2020-01-28	95	62	1	2020-03-16	730	388	0	2020-05-03	342	365	0
2020-01-29	106	74	1	2020-03-17	873	600	2	2020-05-04	528	442	0
2020-01-30	100	74	0	2020-03-18	1197	786	1	2020-05-05	632	660	0
2020-01-31	93	82	0	2020-03-19	1363	1095	3	2020-05-06	451	507	0
2020-02-01	87	76	0	2020-03-20	1371	1236	0	2020-05-07	417	457	0
2020-02-02	64	57	0	2020-03-21	1047	880	3	2020-05-08	402	408	0
2020-02-03	131	116	0	2020-03-22	811	823	1	2020-05-09	269	386	0
2020-02-04	103	91	0	2020-03-23	1139	734	0	2020-05-10	267	293	0
2020-02-05	157	147	0	2020-03-24	924	697	1	2020-05-11	362	307	0
2020-02-06	77	73	0	2020-03-25	938	603	2	2020-05-12	349	343	0
2020-02-07	199	191	0	2020-03-26	929	802	0	2020-05-13	277	317	0
2020-02-08	225	221	0	2020-03-27	864	881	2	2020-05-14	274	285	0
2020-02-09	128	121	0	2020-03-28	527	592	1	2020-05-15	323	306	0
2020-02-10	205	195	0	2020-03-29	835	456	1	2020-05-16	220	283	0
2020-02-11	168	162	0	2020-03-30	1023	679	2	2020-05-17	185	214	0
2020-02-12	128	127	0	2020-03-31	924	708	0	2020-05-18	269	235	0
2020-02-13	115	117	0	2020-04-01	1009	990	2	2020-05-19	223	236	0
2020-02-14	72	73	0	2020-04-02	822	775	2	2020-05-20	226	253	0
2020-02-15	89	91	1	2020-04-03	880	854	1	2020-05-21	236	247	0
2020-02-16	112	115	1	2020-04-04	864	894	0	2020-05-22	187	221	0
2020-02-17	813	827	2	2020-04-05	912	850	1	2020-05-23	170	200	0
2020-02-18	832	841	1	2020-04-06	1814	894	0	2020-05-24	183	183	0
2020-02-19	696	706	1	2020-04-07	1685	1507	1	2020-05-25	222	198	0
2020-02-20	796	808	2	2020-04-08	1628	1725	1	2020-05-26	299	204	0
2020-02-21	714	728	0	2020-04-09	1576	1526	0	2020-05-27	183	286	0
2020-02-22	444	459	2	2020-04-10	1564	1613	0	2020-05-28	170	174	0
2020-02-23	328	340	2	2020-04-11	1091	1288	1	2020-05-29	156	169	0
2020-02-24	698	725	1	2020-04-12	664	1026	0	2020-05-30	174	168	0
2020-02-25	609	632	1	2020-04-13	1340	2183	0	2020-05-31	237	160	0
2020-02-26	521	541	0	2020-04-14	1204	1439	0	2020-06-01	186	197	0
2020-02-27	520	541	1	2020-04-15	952	1078	0	2020-06-02	178	245	0
2020-02-28	371	386	4	2020-04-16	903	1273	0	2020-06-03	188	185	0
2020-02-29	511	513	0	2020-04-17	838	1046	0	2020-06-04	170	175	0
2020-03-01	430	415	1	2020-04-18	557	893	0	2020-06-05	184	201	0
2020-03-02	600	559	1	2020-04-19	624	600	0	2020-06-06	138	177	0

Table 5. The data used for forecasting (continued)

Date	Susp.	Excl.	Conf.	Date	Susp.	Excl.	Conf	Date	Susp.	Excl.	Conf
2020-06-07	108	71	0	2020-07-25	95	85	0	2020-09-11	186	205	0
2020-06-08	129	84	0	2020-07-26	123	112	0	2020-09-12	126	123	0
2020-06-09	152	101	0	2020-07-27	201	182	0	2020-09-13	210	205	0
2020-06-10	152	101	0	2020-07-28	341	143	0	2020-09-14	154	152	0
2020-06-11	135	141	0	2020-07-29	220	320	0	2020-09-15	160	137	0
2020-06-12	142	148	0	2020-07-30	203	232	0	2020-09-16	282	244	0
2020-06-13	118	125	0	2020-07-31	235	271	0	2020-09-17	680	177	0
2020-06-14	111	117	0	2020-08-01	104	120	1	2020-09-18	351	793	0
2020-06-15	145	152	0	2020-08-02	132	196	0	2020-09-19	95	216	0
2020-06-16	147	134	0	2020-08-03	273	178	0	2020-09-20	98	204	0
2020-06-17	146	134	0	2020-08-04	192	126	0	2020-09-21	283	89	0
2020-06-18	135	164	0	2020-08-05	212	215	0	2020-09-22	175	174	0
2020-06-19	159	152	0	2020-08-06	214	182	0	2020-09-23	257	256	0
2020-06-20	114	109	0	2020-08-07	164	200	0	2020-09-24	198	293	0
2020-06-21	104	99	0	2020-08-08	173	212	0	2020-09-25	137	203	0
2020-06-22	156	149	0	2020-08-09	142	194	0	2020-09-26	275	235	0
2020-06-23	126	121	0	2020-08-10	247	230	0	2020-09-27	204	175	0
2020-06-24	173	166	0	2020-08-11	147	137	0	2020-09-28	259	222	0
2020-06-25	217	129	0	2020-08-12	190	178	0	2020-09-29	211	176	0
2020-06-26	176	188	0	2020-08-13	229	235	0	2020-09-30	260	218	0
2020-06-27	154	164	0	2020-08-14	198	204	0	2020-10-01	152	176	0
2020-06-28	127	135	0	2020-08-15	133	137	0	2020-10-02	392	187	0
2020-06-29	184	198	0	2020-08-16	124	180	0	2020-10-03	109	121	0
2020-06-30	210	225	0	2020-08-17	260	116	0	2020-10-04	167	187	0
2020-07-01	141	151	0	2020-08-18	165	216	0	2020-10-05	356	398	0
2020-07-02	129	143	0	2020-08-19	222	204	0	2020-10-06	170	174	0
2020-07-03	140	157	0	2020-08-20	214	236	0	2020-10-07	432	242	0
2020-07-04	119	118	0	2020-08-21	217	239	0	2020-10-08	590	457	0
2020-07-05	151	149	0	2020-08-22	128	142	0	2020-10-09	339	420	0
2020-07-06	177	175	0	2020-08-23	117	111	0	2020-10-10	114	184	0
2020-07-07	136	135	0	2020-08-24	242	230	0	2020-10-11	123	199	0
2020-07-08	141	140	0	2020-08-25	155	147	0	2020-10-12	305	492	0
2020-07-09	188	144	0	2020-08-26	182	173	0	2020-10-13	271	316	0
2020-07-10	165	128	0	2020-08-27	204	210	0	2020-10-14	345	267	0
2020-07-11	118	126	0	2020-08-28	177	183	0	2020-10-15	589	282	0
2020-07-12	126	133	0	2020-08-29	148	153	0	2020-10-16	382	365	0
2020-07-13	126	134	0	2020-08-30	157	166	0	2020-10-17	132	195	0
2020-07-14	155	166	0	2020-08-31	215	229	0	2020-10-18	176	262	0
2020-07-15	164	175	0	2020-09-01	144	153	0	2020-10-19	314	467	0
2020-07-16	191	152	0	2020-09-02	152	162	0	2020-10-20	223	209	0
2020-07-17	145	196	0	2020-09-03	232	181	0	2020-10-21	215	180	0
2020-07-18	111	107	0	2020-09-04	242	189	0	2020-10-22	329	214	0
2020-07-19	135	129	0	2020-09-05	112	186	0	2020-10-23	177	353	0
2020-07-20	161	155	0	2020-09-06	173	204	0	2020-10-24	97	195	0
2020-07-21	143	137	0	2020-09-07	188	91	0	2020-10-25	118	103	0
2020-07-22	156	151	0	2020-09-08	187	221	0	2020-10-26	293	256	0
2020-07-23	163	158	0	2020-09-09	155	185	0	2020-10-27	206	181	0
2020-07-24	135	130	0	2020-09-10	224	146	0	2020-10-28	198	174	0

Table 6. The data used for forecasting (continued)

Date	Susp.	Excl.	Conf.	Date	Susp.	Excl.	Conf	Date	Susp.	Excl.	Conf
2020-10-29	569	192	0	2020-12-16	757	844	0	2021-02-02	3243	1000	0
2020-10-30	156	244	0	2020-12-17	790	715	0	2021-02-03	1167	2029	0
2020-10-31	93	208	0	2020-12-18	577	573	0	2021-02-04	927	1082	0
2020-11-01	116	191	0	2020-12-19	240	502	0	2021-02-05	877	2127	1
2020-11-02	352	237	0	2020-12-20	183	559	0	2021-02-06	517	1918	0
2020-11-03	288	262	0	2020-12-21	760	432	0	2021-02-07	793	825	0
2020-11-04	325	259	0	2020-12-22	800	455	1	2021-02-08	1107	457	0
2020-11-05	272	262	0	2020-12-23	670	851	0	2021-02-09	784	844	1
2020-11-06	394	230	0	2020-12-24	728	560	0	2021-02-10	379	729	0
2020-11-07	114	209	0	2020-12-25	759	586	0	2021-02-11	275	639	0
2020-11-08	118	217	0	2020-12-26	324	678	0	2021-02-12	276	369	0
2020-11-09	320	180	0	2020-12-27	365	624	0	2021-02-13	324	0	0
2020-11-10	292	262	0	2020-12-28	1017	356	0	2021-02-14	334	0	0
2020-11-11	287	388	0	2020-12-29	810	770	0	2021-02-15	325	0	0
2020-11-12	380	363	0	2020-12-30	674	778	0	2021-02-16	320	0	0
2020-11-13	204	296	0	2020-12-31	507	745	0	2021-02-17	759	2001	0
2020-11-14	98	201	0	2021-01-01	200	621	0	2021-02-18	698	632	0
2020-11-15	102	149	0	2021-01-02	251	402	0	2021-02-19	839	821	0
2020-11-16	382	182	0	2021-01-03	159	302	0	2021-02-20	371	839	0
2020-11-17	215	238	0	2021-01-04	1185	180	0	2021-02-21	231	0	0
2020-11-18	287	286	0	2021-01-05	597	654	0	2021-02-22	736	0	0
2020-11-19	429	277	0	2021-01-06	807	987	0	2021-02-23	487	0	0
2020-11-20	199	265	0	2021-01-07	699	603	0	2021-02-24	546	2445	0
2020-11-21	210	325	0	2021-01-08	597	728	0	2021-02-25	544	525	0
2020-11-22	126	195	0	2021-01-09	249	539	0	2021-02-26	418	0	0
2020-11-23	341	200	0	2021-01-10	276	176	0	2021-02-27	221	669	0
2020-11-24	342	240	0	2021-01-11	985	628	0	2021-02-28	197	307	0
2020-11-25	329	232	0	2021-01-12	612	697	2	2021-03-01	276	0	0
2020-11-26	425	320	0	2021-01-13	758	862	0	2021-03-02	434	0	0
2020-11-27	638	392	0	2021-01-14	967	0	0	2021-03-03	288	789	0
2020-11-28	479	359	0	2021-01-15	1174	1446	0	2021-03-04	675	278	0
2020-11-29	241	693	0	2021-01-16	543	836	0	2021-03-05	706	0	0
2020-11-30	337	377	0	2021-01-17	447	854	2	2021-03-06	349	1225	0
2020-12-01	363	280	0	2021-01-18	1518	610	1	2021-03-07	176	771	0
2020-12-02	184	262	0	2021-01-19	951	657	4	2021-03-08	1093	233	0
2020-12-03	898	301	0	2021-01-20	1244	1178	1	2021-03-09	357	551	0
2020-12-04	283	280	0	2021-01-21	1188	1202	0	2021-03-10	490	880	0
2020-12-05	146	605	0	2021-01-22	1324	1341	2	2021-03-11	626	0	0
2020-12-06	137	352	0	2021-01-23	1007	1081	1	2021-03-12	641	799	0
2020-12-07	242	141	0	2021-01-24	593	1293	2	2021-03-13	212	0	0
2020-12-08	291	170	0	2021-01-25	1738	0	0	2021-03-14	142	1034	0
2020-12-09	300	211	0	2021-01-26	1516	1867	0	2021-03-15	536	152	0
2020-12-10	434	78	0	2021-01-27	1629	1639	0	2021-03-16	650	0	0
2020-12-11	780	330	0	2021-01-28	1602	1290	0	2021-03-17	485	865	0
2020-12-12	423	540	0	2021-01-29	1878	1609	0	2021-03-18	360	479	0
2020-12-13	216	1065	0	2021-01-30	1359	1362	4	2021-03-19	432	713	0
2020-12-14	847	270	0	2021-01-31	872	1277	0	2021-03-20	219	424	0
2020-12-15	801	594	0	2021-02-01	1210	1220	0	2021-03-21	163	391	0

Table 7. The data used for forecasting (continued)

Date	Susp.	Excl.	Conf.	Date	Susp.	Excl.	Conf	Date	Susp.	Excl.	Conf
2021-03-22	803	0	0	2021-04-20	526	413	2	2021-05-19	17528	8746	265
2021-03-23	437	771	0	2021-04-21	837	577	0	2021-05-20	17892	12403	283
2021-03-24	569	706	0	2021-04-22	670	520	0	2021-05-21	21653	15358	311
2021-03-25	716	556	0	2021-04-23	832	920	2	2021-05-22	14450	17597	721
2021-03-26	628	763	0	2021-04-24	543	656	1	2021-05-23	12865	14176	457
2021-03-27	252	637	0	2021-04-25	614	479	2	2021-05-24	22926	12381	590
2021-03-28	164	405	0	2021-04-26	1099	488	2	2021-05-25	24383	15368	537
2021-03-29	850	212	0	2021-04-27	752	1089	1	2021-05-26	20871	18701	633
2021-03-30	834	604	0	2021-04-28	764	1018	3	2021-05-27	19811	22596	666
2021-03-31	964	807	0	2021-04-29	1144	685	3	2021-05-28	28993	15799	552
2021-04-01	604	623	0	2021-04-30	854	1056	3	2021-05-29	16141	29381	484
2021-04-02	275	693	0	2021-05-01	383	766	1	2021-05-30	13535	27136	354
2021-04-03	290	412	0	2021-05-02	418	625	4	2021-05-31	27502	11967	347
2021-04-04	158	362	0	2021-05-03	1393	435	2	2021-06-01	30905	22678	326
2021-04-05	303	171	0	2021-05-04	988	993	2	2021-06-02	29598	35689	548
2021-04-06	1159	421	0	2021-05-05	1094	1194	0	2021-06-03	27796	31670	583
2021-04-07	658	478	0	2021-05-06	1006	1001	1	2021-06-04	31416	29217	470
2021-04-08	669	0	0	2021-05-07	1067	939	0	2021-06-05	18505	29470	510
2021-04-09	846	1716	0	2021-05-08	413	926	2	2021-06-06	13167	20020	342
2021-04-10	230	658	0	2021-05-09	680	698	0	2021-06-07	33272	14079	190
2021-04-11	159	553	0	2021-05-10	1237	556	4	2021-06-08	31728	26995	203
2021-04-12	755	344	0	2021-05-11	1218	1003	7	2021-06-09	33580	41180	273
2021-04-13	538	638	0	2021-05-12	2432	1136	17	2021-06-10	35025	29801	251
2021-04-14	598	721	0	2021-05-13	3893	1647	13	2021-06-11	36148	34517	277
2021-04-15	526	570	0	2021-05-14	6177	0	29	2021-06-12	22323	40622	245
2021-04-16	548	507	0	2021-05-15	6497	7736	180	2021-06-13	15821	24841	174
2021-04-17	139	511	0	2021-05-16	8425	4755	206	2021-06-14	19907	16906	185
2021-04-18	128	319	0	2021-05-17	15447	6322	333	2021-06-15	30647	17792	132
2021-04-19	719	133	0	2021-05-18	18087	8306	238	2021-06-16	25689	33366	165

Table 8. The updated data used for refitting

Date	Susp.	Excl.	Conf.	Date	Susp.	Excl.	Conf	Date	Susp.	Excl.	Conf
2020-01-15	0	0	0	2020-03-03	506	466	0	2020-04-20	1835	931	0
2020-01-16	4	2	0	2020-03-04	485	454	1	2020-04-21	1371	1526	0
2020-01-17	0	0	0	2020-03-05	297	283	1	2020-04-22	1154	1469	0
2020-01-18	1	0	0	2020-03-06	381	393	0	2020-04-23	1014	1163	0
2020-01-19	1	0	0	2020-03-07	307	382	0	2020-04-24	827	1132	0
2020-01-20	2	1	0	2020-03-08	298	313	0	2020-04-25	619	1091	0
2020-01-21	12	5	0	2020-03-09	476	359	1	2020-04-26	495	738	0
2020-01-22	44	7	0	2020-03-10	332	376	0	2020-04-27	740	588	0
2020-01-23	70	14	0	2020-03-11	445	395	0	2020-04-28	694	792	0
2020-01-24	57	20	0	2020-03-12	677	685	1	2020-04-29	484	705	0
2020-01-25	91	42	0	2020-03-13	439	454	0	2020-04-30	495	513	0
2020-01-26	117	58	0	2020-03-14	379	405	0	2020-05-01	374	477	0
2020-01-27	123	64	0	2020-03-15	306	341	0	2020-05-02	380	436	0
2020-01-28	95	62	1	2020-03-16	730	388	0	2020-05-03	342	365	0
2020-01-29	106	74	1	2020-03-17	873	600	2	2020-05-04	527	442	0
2020-01-30	100	74	0	2020-03-18	1197	786	1	2020-05-05	632	660	0
2020-01-31	93	82	0	2020-03-19	1363	1095	3	2020-05-06	451	507	0
2020-02-01	87	76	0	2020-03-20	1371	1236	0	2020-05-07	417	457	0
2020-02-02	64	57	0	2020-03-21	1047	880	3	2020-05-08	402	408	0
2020-02-03	131	116	0	2020-03-22	811	823	1	2020-05-09	269	386	0
2020-02-04	103	91	0	2020-03-23	1139	734	0	2020-05-10	267	293	0
2020-02-05	157	147	0	2020-03-24	924	697	1	2020-05-11	362	307	0
2020-02-06	77	73	0	2020-03-25	938	603	2	2020-05-12	349	343	0
2020-02-07	199	191	0	2020-03-26	929	802	0	2020-05-13	277	317	0
2020-02-08	225	221	0	2020-03-27	864	881	2	2020-05-14	274	285	0
2020-02-09	128	121	0	2020-03-28	527	592	1	2020-05-15	323	306	0
2020-02-10	205	195	0	2020-03-29	835	456	1	2020-05-16	220	283	0
2020-02-11	168	162	0	2020-03-30	1023	679	2	2020-05-17	185	214	0
2020-02-12	128	127	0	2020-03-31	924	708	0	2020-05-18	269	235	0
2020-02-13	115	117	0	2020-04-01	1009	990	2	2020-05-19	223	236	0
2020-02-14	72	73	0	2020-04-02	822	775	2	2020-05-20	226	253	0
2020-02-15	89	91	1	2020-04-03	880	854	1	2020-05-21	236	247	0
2020-02-16	112	115	1	2020-04-04	864	894	0	2020-05-22	187	221	0
2020-02-17	813	827	2	2020-04-05	912	850	1	2020-05-23	170	200	0
2020-02-18	832	841	1	2020-04-06	1814	894	0	2020-05-24	183	183	0
2020-02-19	696	706	1	2020-04-07	1685	1507	1	2020-05-25	222	198	0
2020-02-20	796	808	2	2020-04-08	1628	1725	1	2020-05-26	299	204	0
2020-02-21	714	728	0	2020-04-09	1576	1526	0	2020-05-27	183	286	0
2020-02-22	444	459	2	2020-04-10	1564	1613	0	2020-05-28	170	174	0
2020-02-23	328	340	2	2020-04-11	1091	1288	1	2020-05-29	156	169	0
2020-02-24	698	725	1	2020-04-12	664	1026	0	2020-05-30	174	168	0
2020-02-25	609	632	1	2020-04-13	1340	2183	0	2020-05-31	237	160	0
2020-02-26	521	541	0	2020-04-14	1204	1439	0	2020-06-01	186	197	0
2020-02-27	520	541	1	2020-04-15	952	1078	0	2020-06-02	178	245	0
2020-02-28	371	386	4	2020-04-16	903	1273	0	2020-06-03	188	185	0
2020-02-29	511	513	0	2020-04-17	838	1046	0	2020-06-04	170	175	0
2020-03-01	430	415	1	2020-04-18	557	893	0	2020-06-05	184	201	0
2020-03-02	600	559	1	2020-04-19	624	600	0	2020-06-06	138	177	0

Table 9. The updated data used for refitting (continued)

Date	Susp.	Excl.	Conf.	Date	Susp.	Excl.	Conf	Date	Susp.	Excl.	Conf
2020-06-07	108	71	0	2020-07-25	95	85	0	2020-09-11	186	480	0
2020-06-08	129	84	0	2020-07-26	123	112	0	2020-09-12	126	97	0
2020-06-09	152	101	0	2020-07-27	201	182	0	2020-09-13	210	164	0
2020-06-10	152	101	0	2020-07-28	341	143	0	2020-09-14	154	120	0
2020-06-11	135	141	0	2020-07-29	220	320	0	2020-09-15	160	64	0
2020-06-12	142	148	0	2020-07-30	203	232	0	2020-09-16	282	113	0
2020-06-13	118	125	0	2020-07-31	235	271	0	2020-09-17	680	1009	0
2020-06-14	111	117	0	2020-08-01	104	120	1	2020-09-18	351	160	0
2020-06-15	145	152	0	2020-08-02	132	196	0	2020-09-19	95	44	0
2020-06-16	147	134	0	2020-08-03	273	178	0	2020-09-20	98	89	0
2020-06-17	146	134	0	2020-08-04	192	126	0	2020-09-21	283	430	0
2020-06-18	135	164	0	2020-08-05	211	215	0	2020-09-22	175	200	0
2020-06-19	159	152	0	2020-08-06	214	182	0	2020-09-23	257	296	0
2020-06-20	114	109	0	2020-08-07	164	200	0	2020-09-24	198	374	0
2020-06-21	104	99	0	2020-08-08	173	212	0	2020-09-25	137	258	0
2020-06-22	156	149	0	2020-08-09	142	194	0	2020-09-26	275	147	0
2020-06-23	126	121	0	2020-08-10	247	230	0	2020-09-27	204	108	0
2020-06-24	173	166	0	2020-08-11	147	137	0	2020-09-28	259	139	0
2020-06-25	217	129	0	2020-08-12	190	178	0	2020-09-29	211	78	0
2020-06-26	176	188	0	2020-08-13	229	235	0	2020-09-30	260	98	0
2020-06-27	154	164	0	2020-08-14	198	204	0	2020-10-01	152	187	0
2020-06-28	127	135	0	2020-08-15	133	137	0	2020-10-02	392	706	0
2020-06-29	184	198	0	2020-08-16	124	180	0	2020-10-03	109	29	0
2020-06-30	210	225	0	2020-08-17	260	116	0	2020-10-04	167	46	0
2020-07-01	141	151	0	2020-08-18	165	216	0	2020-10-05	356	99	0
2020-07-02	129	143	0	2020-08-19	222	204	0	2020-10-06	170	242	0
2020-07-03	140	157	0	2020-08-20	214	236	0	2020-10-07	432	457	0
2020-07-04	119	118	0	2020-08-21	217	239	0	2020-10-08	590	420	0
2020-07-05	151	149	0	2020-08-22	128	142	0	2020-10-09	339	875	0
2020-07-06	177	175	0	2020-08-23	117	111	0	2020-10-10	114	66	0
2020-07-07	136	135	0	2020-08-24	242	230	0	2020-10-11	123	72	0
2020-07-08	141	140	0	2020-08-25	155	147	0	2020-10-12	305	178	0
2020-07-09	188	144	0	2020-08-26	182	173	0	2020-10-13	271	267	0
2020-07-10	165	128	0	2020-08-27	204	210	0	2020-10-14	345	282	0
2020-07-11	118	126	0	2020-08-28	177	183	0	2020-10-15	589	365	0
2020-07-12	126	133	0	2020-08-29	148	153	0	2020-10-16	382	924	0
2020-07-13	126	134	0	2020-08-30	157	166	0	2020-10-17	132	44	0
2020-07-14	155	166	0	2020-08-31	215	229	0	2020-10-18	176	59	0
2020-07-15	164	175	0	2020-09-01	144	153	0	2020-10-19	314	106	0
2020-07-16	191	152	0	2020-09-02	152	162	0	2020-10-20	223	180	0
2020-07-17	145	196	0	2020-09-03	232	181	0	2020-10-21	215	214	0
2020-07-18	111	107	0	2020-09-04	242	189	0	2020-10-22	329	548	0
2020-07-19	135	129	0	2020-09-05	112	186	0	2020-10-23	177	461	0
2020-07-20	161	155	0	2020-09-06	173	204	0	2020-10-24	97	253	0
2020-07-21	143	137	0	2020-09-07	188	91	0	2020-10-25	118	27	0
2020-07-22	156	151	0	2020-09-08	187	221	0	2020-10-26	293	69	0
2020-07-23	163	158	0	2020-09-09	155	185	0	2020-10-27	206	49	0
2020-07-24	135	130	0	2020-09-10	224	351	0	2020-10-28	198	47	0

Table 10. The updated data used for refitting (continued)

Date	Susp.	Excl.	Conf.	Date	Susp.	Excl.	Conf	Date	Susp.	Excl.	Conf
2020-10-29	569	244	0	2020-12-16	757	715	0	2021-02-02	3243	2029	0
2020-10-30	156	208	0	2020-12-17	790	573	0	2021-02-03	1167	1082	0
2020-10-31	93	191	0	2020-12-18	577	502	0	2021-02-04	927	2127	1
2020-11-01	116	237	0	2020-12-19	240	559	0	2021-02-05	877	1918	0
2020-11-02	352	262	0	2020-12-20	183	887	1	2021-02-06	517	825	0
2020-11-03	288	259	0	2020-12-21	760	414	0	2021-02-07	791	457	0
2020-11-04	325	262	0	2020-12-22	800	437	0	2021-02-08	1106	844	1
2020-11-05	272	230	0	2020-12-23	670	1146	0	2021-02-09	783	729	0
2020-11-06	394	426	0	2020-12-24	728	332	0	2021-02-10	378	639	0
2020-11-07	114	88	0	2020-12-25	759	346	0	2021-02-11	275	369	0
2020-11-08	118	92	0	2020-12-26	324	624	0	2021-02-12	276	0	0
2020-11-09	320	262	0	2020-12-27	365	356	0	2021-02-13	324	0	0
2020-11-10	292	388	0	2020-12-28	1017	770	0	2021-02-14	334	0	0
2020-11-11	287	363	0	2020-12-29	810	778	0	2021-02-15	325	0	0
2020-11-12	380	296	0	2020-12-30	674	745	0	2021-02-16	320	2001	0
2020-11-13	204	201	0	2020-12-31	507	621	0	2021-02-17	758	632	0
2020-11-14	98	149	0	2021-01-01	200	402	0	2021-02-18	698	821	0
2020-11-15	102	182	0	2021-01-02	251	302	0	2021-02-19	839	839	0
2020-11-16	382	238	0	2021-01-03	159	180	0	2021-02-20	371	0	0
2020-11-17	215	286	0	2021-01-04	1185	654	0	2021-02-21	231	0	0
2020-11-18	287	277	0	2021-01-05	597	987	0	2021-02-22	735	0	0
2020-11-19	429	265	0	2021-01-06	807	603	0	2021-02-23	487	2445	0
2020-11-20	199	520	0	2021-01-07	699	728	0	2021-02-24	546	525	0
2020-11-21	210	125	0	2021-01-08	597	539	0	2021-02-25	544	0	0
2020-11-22	126	75	0	2021-01-09	249	804	0	2021-02-26	417	669	0
2020-11-23	341	472	0	2021-01-10	276	152	1	2021-02-27	221	307	0
2020-11-24	342	163	0	2021-01-11	985	545	1	2021-02-28	197	0	0
2020-11-25	329	157	0	2021-01-12	612	862	0	2021-03-01	276	0	0
2020-11-26	425	392	0	2021-01-13	758	0	0	2021-03-02	434	789	0
2020-11-27	638	359	0	2021-01-14	967	1446	0	2021-03-03	288	278	0
2020-11-28	479	693	0	2021-01-15	1174	836	0	2021-03-04	675	0	0
2020-11-29	241	377	0	2021-01-16	543	854	2	2021-03-05	706	1225	0
2020-11-30	337	280	0	2021-01-17	447	610	1	2021-03-06	349	771	0
2020-12-01	363	262	0	2021-01-18	1518	657	4	2021-03-07	176	233	0
2020-12-02	184	301	0	2021-01-19	949	1178	1	2021-03-08	1093	551	0
2020-12-03	898	280	0	2021-01-20	1244	1202	0	2021-03-09	357	880	0
2020-12-04	283	605	0	2021-01-21	1187	1341	2	2021-03-10	490	0	0
2020-12-05	146	352	0	2021-01-22	1323	1081	1	2021-03-11	626	799	0
2020-12-06	137	311	0	2021-01-23	1007	1293	2	2021-03-12	641	0	0
2020-12-07	242	95	0	2021-01-24	593	0	0	2021-03-13	212	1034	0
2020-12-08	291	116	0	2021-01-25	1735	1867	0	2021-03-14	142	152	0
2020-12-09	300	78	0	2021-01-26	1516	1639	0	2021-03-15	535	0	0
2020-12-10	434	330	0	2021-01-27	1629	1290	0	2021-03-16	649	865	0
2020-12-11	780	540	0	2021-01-28	1601	1609	0	2021-03-17	485	479	0
2020-12-12	423	1065	0	2021-01-29	1877	1362	4	2021-03-18	360	713	0
2020-12-13	216	270	0	2021-01-30	1358	1277	0	2021-03-19	432	424	0
2020-12-14	847	594	0	2021-01-31	872	1220	0	2021-03-20	219	391	0
2020-12-15	801	844	0	2021-02-01	1210	1000	0	2021-03-21	163	0	0

Table 11. The updated data used for refitting (continued)

Date	Susp.	Excl.	Conf.	Date	Susp.	Excl.	Conf	Date	Susp.	Excl.	Conf
2021-03-22	803	771	0	2021-05-09	680	556	4	2021-06-26	20093	23115	88
2021-03-23	437	706	0	2021-05-10	1237	1003	7	2021-06-27	14241	14841	60
2021-03-24	568	556	0	2021-05-11	1217	1136	17	2021-06-28	37365	30109	54
2021-03-25	716	763	0	2021-05-12	2421	1647	13	2021-06-29	34428	35516	54
2021-03-26	628	637	0	2021-05-13	3891	0	29	2021-06-30	30914	32076	46
2021-03-27	251	405	0	2021-05-14	6141	7736	180	2021-07-01	29791	32041	57
2021-03-28	164	212	0	2021-05-15	6496	4755	206	2021-07-02	28066	25551	76
2021-03-29	849	604	0	2021-05-16	8395	6322	333	2021-07-03	19341	21940	37
2021-03-30	834	807	0	2021-05-17	15432	8306	238	2021-07-04	16410	13034	26
2021-03-31	965	623	0	2021-05-18	18072	8746	265	2021-07-05	33423	31048	25
2021-04-01	604	693	0	2021-05-19	17514	12403	283	2021-07-06	26399	28966	39
2021-04-02	275	412	0	2021-05-20	17881	15358	311	2021-07-07	26895	9387	18
2021-04-03	290	362	0	2021-05-21	21603	17597	721	2021-07-08	24427	41064	32
2021-04-04	158	171	0	2021-05-22	14429	14176	457	2021-07-09	23405	23333	31
2021-04-05	303	421	0	2021-05-23	12854	12381	590	2021-07-10	16941	18933	28
2021-04-06	1159	478	0	2021-05-24	22906	15368	537	2021-07-11	13527	14132	23
2021-04-07	658	0	0	2021-05-25	24359	18701	633	2021-07-12	27848	21852	28
2021-04-08	669	1716	0	2021-05-26	20843	22596	666	2021-07-13	22985	25115	16
2021-04-09	846	658	0	2021-05-27	19783	15799	552	2021-07-14	22455	20340	14
2021-04-10	230	553	0	2021-05-28	30390	29381	484	2021-07-15	18515	20833	29
2021-04-11	159	344	0	2021-05-29	18535	27136	354	2021-07-16	23470	19772	7
2021-04-12	755	638	0	2021-05-30	12849	11967	347	2021-07-17	15897	17068	15
2021-04-13	538	721	0	2021-05-31	28428	22678	326	2021-07-18	12576	14065	15
2021-04-14	597	570	0	2021-06-01	28933	35689	548	2021-07-19	27544	22880	18
2021-04-15	526	507	0	2021-06-02	29076	31670	583	2021-07-20	24259	26769	16
2021-04-16	547	511	0	2021-06-03	28436	29217	470	2021-07-21	25214	24151	30
2021-04-17	139	319	0	2021-06-04	30936	29470	510	2021-07-22	19646	23485	23
2021-04-18	128	133	0	2021-06-05	19504	20020	342	2021-07-23	20521	18982	22
2021-04-19	719	413	2	2021-06-06	15371	14079	190	2021-07-24	14628	17231	12
2021-04-20	525	577	0	2021-06-07	34337	26995	203	2021-07-25	13083	12237	10
2021-04-21	836	520	0	2021-06-08	31911	41180	273	2021-07-26	28813	24816	16
2021-04-22	670	920	2	2021-06-09	33773	29801	251	2021-07-27	25039	26138	18
2021-04-23	831	656	1	2021-06-10	34318	34517	277	2021-07-28	23667	22741	16
2021-04-24	543	479	2	2021-06-11	37334	40622	245	2021-07-29	20570	23219	21
2021-04-25	613	488	2	2021-06-12	21908	24841	174	2021-07-30	25805	21598	11
2021-04-26	1099	1089	1	2021-06-13	15826	16906	185	2021-07-31	17523	20132	12
2021-04-27	752	1018	3	2021-06-14	19201	17792	132	2021-08-01	13009	14200	12
2021-04-28	764	685	3	2021-06-15	30717	33366	165	2021-08-02	28420	23504	16
2021-04-29	1143	1056	3	2021-06-16	33851	36144	175	2021-08-03	24291	27307	20
2021-04-30	854	766	1	2021-06-17	36908	42005	186	2021-08-04	23332	24102	6
2021-05-01	383	625	4	2021-06-18	32326	35888	124	2021-08-05	18360	18485	10
2021-05-02	418	435	2	2021-06-19	18348	19428	107	2021-08-06	22651	20631	5
2021-05-03	1391	993	2	2021-06-20	13986	13298	75	2021-08-07	14345	16570	4
2021-05-04	988	1194	0	2021-06-21	32397	30348	76	2021-08-08	12554	13010	4
2021-05-05	1094	1001	1	2021-06-22	34802	41268	103	2021-08-09	26434	22415	3
2021-05-06	1005	939	0	2021-06-23	32464	30986	128	2021-08-10	23073	24543	12
2021-05-07	1066	926	2	2021-06-24	33017	33506	76	2021-08-11	22490	21042	4
2021-05-08	413	698	0	2021-06-25	30043	30016	78	2021-08-12	18063	20361	2

Table 12. The updated data used for refitting (continued)

Date	Susp.	Excl.	Conf.	Date	Susp.	Excl.	Conf	Date	Susp.	Excl.	Conf
2021-08-13	22746	20430	3	2021-09-30	19966	21310	0	2021-11-17	17310	17499	0
2021-08-14	16304	18152	2	2021-10-01	23717	21599	0	2021-11-18	14309	15397	0
2021-08-15	12754	12678	8	2021-10-02	17970	19671	0	2021-11-19	17615	16062	0
2021-08-16	25879	22196	4	2021-10-03	12992	14336	0	2021-11-20	13060	7898	0
2021-08-17	23029	23762	6	2021-10-04	27757	23315	0	2021-11-21	10339	19254	0
2021-08-18	23158	22305	1	2021-10-05	23234	24687	0	2021-11-22	20075	16063	0
2021-08-19	19800	21320	6	2021-10-06	22552	22633	0	2021-11-23	16754	17797	0
2021-08-20	21793	21803	2	2021-10-07	18369	20731	0	2021-11-24	16576	16410	0
2021-08-21	15884	16695	6	2021-10-08	21336	19717	1	2021-11-25	14251	14854	0
2021-08-22	11999	13434	4	2021-10-09	16724	18267	0	2021-11-26	18674	16473	0
2021-08-23	26142	22253	1	2021-10-10	11962	13046	1	2021-11-27	12834	14772	0
2021-08-24	23756	24468	0	2021-10-11	19801	16615	0	2021-11-28	9804	13855	0
2021-08-25	24118	24412	2	2021-10-12	25772	25546	0	2021-11-29	22315	17287	0
2021-08-26	20243	23296	0	2021-10-13	21860	23036	0	2021-11-30	17369	18723	0
2021-08-27	23275	20801	0	2021-10-14	17435	20118	0	2021-12-01	17082	15849	0
2021-08-28	17483	19276	13	2021-10-15	23171	19513	1	2021-12-02	13402	15184	0
2021-08-29	13036	13321	3	2021-10-16	16230	17126	0	2021-12-03	18128	16150	0
2021-08-30	32010	27803	3	2021-10-17	11090	36611	1	2021-12-04	12269	13223	0
2021-08-31	28547	29794	0	2021-10-18	23697	10570	0	2021-12-05	10161	9960	0
2021-09-01	25584	25425	1	2021-10-19	19059	10570	0	2021-12-06	20873	19813	0
2021-09-02	20839	22961	2	2021-10-20	19969	18975	0	2021-12-07	17229	18293	0
2021-09-03	27122	22354	0	2021-10-21	15540	17700	2	2021-12-08	16858	16087	0
2021-09-04	20119	23120	2	2021-10-22	18633	17672	0	2021-12-09	13872	15529	1
2021-09-05	16385	16942	9	2021-10-23	14048	15921	0	2021-12-10	18450	16345	0
2021-09-06	34709	30730	8	2021-10-24	10175	10461	2	2021-12-11	12339	14488	0
2021-09-07	30583	32134	7	2021-10-25	22507	16921	0	2021-12-12	9488	11069	0
2021-09-08	29855	29843	4	2021-10-26	18368	20326	0	2021-12-13	17820	15683	0
2021-09-09	24870	27132	2	2021-10-27	18419	18619	0	2021-12-14	20626	16824	0
2021-09-10	29325	27261	0	2021-10-28	15463	16854	0	2021-12-15	20560	17721	1
2021-09-11	22050	25199	3	2021-10-29	19083	17328	0	2021-12-16	16038	19631	7
2021-09-12	9892	14917	2	2021-10-30	14697	16297	0	2021-12-17	19423	18299	0
2021-09-13	31057	22754	2	2021-10-31	11066	10739	0	2021-12-18	12807	16391	0
2021-09-14	26359	28449	1	2021-11-01	23559	21638	0	2021-12-19	10555	11892	0
2021-09-15	25134	25370	2	2021-11-02	19227	21089	0	2021-12-20	21507	19442	0
2021-09-16	21348	21627	2	2021-11-03	19029	18797	1	2021-12-21	17305	19277	0
2021-09-17	20163	22197	0	2021-11-04	15515	16728	0	2021-12-22	16206	15574	0
2021-09-18	15376	15318	1	2021-11-05	20457	18400	0	2021-12-23	14164	15291	0
2021-09-19	12892	13037	2	2021-11-06	14742	15826	0	2021-12-24	16658	16056	0
2021-09-20	22756	19531	0	2021-11-07	10700	11344	0	2021-12-25	12848	11758	1
2021-09-21	14319	16808	0	2021-11-08	20343	18438	0	2021-12-26	10457	10874	0
2021-09-22	29230	25740	0	2021-11-09	16880	19174	0	2021-12-27	17684	18236	0
2021-09-23	20624	24208	1	2021-11-10	17297	16808	0	2021-12-28	15195	16291	0
2021-09-24	27852	25588	0	2021-11-11	13983	15286	0	2021-12-29	15129	13763	0
2021-09-25	19848	22393	0	2021-11-12	17362	16368	0	2021-12-30	13792	14803	0
2021-09-26	14698	15841	0	2021-11-13	12888	14688	0	2021-12-31	14391	14798	0
2021-09-27	30711	25144	1	2021-11-14	9809	9953	0	2022-01-01	10338	9557	0
2021-09-28	25129	28789	1	2021-11-15	22351	18688	0	2022-01-02	10022	11032	2
2021-09-29	24493	24371	0	2021-11-16	18260	19468	0	2022-01-03	19298	16781	4
